# A conformational change within the WAVE2 complex regulates its degradation following cellular activation

**DOI:** 10.1038/srep44863

**Published:** 2017-03-23

**Authors:** Noah Joseph, Guy Biber, Sophia Fried, Barak Reicher, Omer Levy, Batel Sabag, Elad Noy, Mira Barda-Saad

**Affiliations:** 1 The Mina and Everard Goodman Faculty of Life Sciences, Bar-Ilan University, Ramat-Gan 5290002, Israel

## Abstract

WASp family Verprolin-homologous protein-2 (WAVE2), a member of the Wiskott-Aldrich syndrome protein (WASp) family of actin nucleation promoting factors, is a central regulator of actin cytoskeleton polymerization and dynamics. Multiple signaling pathways operate via WAVE2 to promote the actin-nucleating activity of the actin-related protein 2/3 (Arp2/3) complex. WAVE2 exists as a part of a pentameric protein complex known as the WAVE regulatory complex (WRC), which is unstable in the absence of its individual proteins. While the involvement of WAVE2 in actin polymerization has been well documented, its negative regulation mechanism is poorly characterized to date. Here, we demonstrate that WAVE2 undergoes ubiquitylation in a T-cell activation dependent manner, followed by proteasomal degradation. The WAVE2 ubiquitylation site was mapped to lysine 45, located at the N-terminus where WAVE2 binds to the WRC. Using Förster resonance energy transfer (FRET), we reveal that the autoinhibitory conformation of the WRC maintains the stability of WAVE2 in resting cells; the release of autoinhibition following T-cell activation facilitates the exposure of WAVE2 to ubiquitylation, leading to its degradation. The dynamic conformational structures of WAVE2 during cellular activation dictate its degradation.

WASp family Verprolin-homologous (WAVE) (also known as SCAR) proteins include three isoforms in mammals, termed WAVE1-3. All family members are fundamental regulators of actin polymerization^1^, required in numerous cellular functions such as the immune response, embryonic development, tissue repair, and cell motility and migration. They are essential mediators of the production and dynamics of most actin-rich protrusions, including pseudopodia, lamellipodia^2^,^3^, and filopodia^4^. WAVE1 and WAVE3 are expressed primarily in neuronal cells, whereas WAVE2 is mainly expressed in cells of the hematopoietic system^5^. Interestingly, WAVE2-deficient mice die during gestation and display defects in development, cell migration, lamellipodia formation and dorsal ruffling, corroborating the critical role of this factor in actin assembly^6^,^7^,^8^,^9^. Actin cytoskeletal reorganization is crucial for T cell activation and plays an important role in T cell spreading, immunological synapse (IS) formation, Ca^2+^ influx and secretion of cytokines and cytolytic granules at the T-cell:antigen presenting cell (APC) contact site^10^,^11^. WAVE2 was identified as a central regulator of F-actin polymerization and rearrangement downstream to the T cell receptor (TCR)^12^,^13^. It was shown that WAVE2 is recruited to the IS, and that RNAi-mediated depletion of WAVE2 inhibits TCR-induced spreading and F-actin polymerization at the IS^12^,^13^. WAVE2 was also found to be involved in integrin-mediated TCR-stimulated adhesion^14^,^15^, Ca^2+^ release-activated Ca^2+^ (CRAC) channels-mediated Ca^2+^ entry, TCR-mediated activation of nuclear factor of activated T cells (NFAT), and is required for TCR-stimulated IL-2 promoter activity^12^,^14^,^16^. These early observations established WAVE2 as an integral component of TCR signaling cascade.

Structurally, WAVE proteins contain a WAVE/SCAR homology domain (WHD/SHD) at their N-terminus, immediately followed by a basic region (B)^17^,^18^,^19^. Adjacent to the B domain, is a proline-rich domain (PRD), which serves as a binding site for proteins containing Src-homology 3 (SH3) domains. The WAVE proteins possess a conserved verprolin-homology cofilin-homology acidic (VCA) domain at their C-terminus, allowing them to stimulate actin nucleation by interacting with both actin monomers and the actin-related protein 2/3 (Arp2/3) complex^17^,^18^,^19^,^20^. This domain must be tightly regulated to ensure proper spatial and temporal control over actin assembly, as dysregulation of actin nucleation can contribute to the pathogenesis of several diseases, such as chronic inflammatory diseases, tumor progression and metastasis^21^,^22^,^23^.

WAVE proteins are constitutively present within a heteropentameric complex, known as the WAVE regulatory complex (WRC), in various organisms, including mammalian cells^24^,^25^,^26^. The entire complex is highly conserved through eukaryotic evolution^27^ and comprises four additional proteins, namely Sra1/PIR121, Nap1/Hem-1, Abi1/2, and HSPC300 at a 1:1:1:1:1 molar ratio^24^,^28^,^29^. WAVE interacts with the WRC complex members mainly at the N-terminus^30^.

The factors regulating the stability of the WRC members are not clear. Previously it was shown by Nolz *et al*. that stability of the WRC components is inter-dependent; elimination of any component results in decreased amounts of WAVE as well as of the other constituents of the complex^12^. However, another study, claimed otherwise, and showed the stability of the entire WAVE complex is unaffected by knockdown of individual components of the complex in T cells^13^. Moreover, it was shown in *Dictyostelium* that cells expressing a mutant SCAR (a WAVE homolog), lacking a WRC binding site, produce a stable protein in both wild type cells and in cells missing various members of the complex^30^. Therefore, our understanding of the integrity of the WRC is incomplete.

Interactions with prenylated Rac-GTP, acidic phospholipids, and protein kinases, such as Abl, were found to be essential for the activation of WAVE2 and its regulatory complex^31^,^32^. These regulators must be present simultaneously, as partial activation is not achieved by any subset of these mediators^31^. In addition, these activators function in a highly cooperative process as they recruit and cluster the WRC at the plasma membrane, leading to the activation of multiple WAVE complexes in close proximity^31^,^33^.

The crystal structure of the human WAVE1 complex^28^ shows that the WRC is composed of a Sra1:Nap1 dimer that forms a platform for a WAVE1:Abi2:HSPC300 trimer. The trimer contacts the dimer in a tripartite manner, through extensive interactions along an axis formed by the dimer. Although the WAVE1 used for the crystal structure determination was deleted of its PRD, it was suggested that its VCA is sequestered in a concave surface formed by Sra1 and residues 82–184 of WAVE1. Thus, WAVE is maintained in an inactive form toward Arp2/3 within the WRC^28^. A conformational change, induced by Rac-GTP binding to Sra1, leads to a release of the sequestered VCA, without dissociating the complex^28^. It is not yet known whether the conformational structure of WAVE1 resembles that of WAVE2, and the role of the PRD in WAVE conformational change has not been elucidated. Furthermore, the molecular link between WAVE2 activation and its degradation remains unknown.

While the positive control mechanisms of WAVE2 regulating its involvement in actin polymerization have been well documented, the pathways resulting in its negative regulation have not been described. In this study, we characterize the molecular linkage between WAVE2 activation and its degradation. We show that ubiquitylation of WAVE2 is dependent on T-cell activation, and is followed by proteasomal degradation. We further map the WAVE2 degradation site to its N-terminal WHD, and specifically, to lysine 45. The WAVE2 complex masks its own degradation site by interaction with the VCA domain, and release of WAVE2 from its autoinhibitory conformation leads to its degradation following cellular activation. Thus our findings demonstrate that regulated degradation of WAVE2 is dependent upon its conformational changes throughout cellular activation.

## Results

### WAVE2 is modified by ubiquitylation induced by TCR activation, marking it for proteasomal degradation

**Figure 1:**
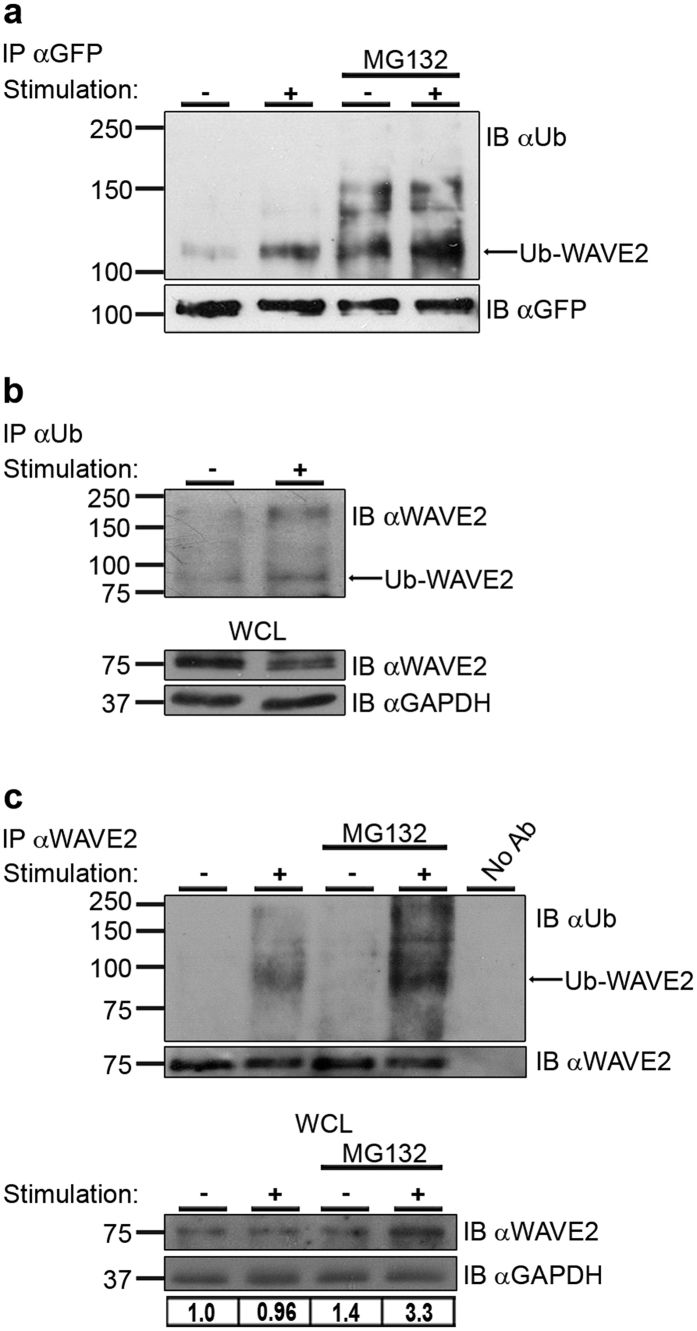
Ubiquitylation of WAVE2. (**a**) Jurkat T cells expressing YFP-WAVE2 were left unstimulated (−), or were co-stimulated with anti-CD3 and anti-CD28 antibodies (+). MG132-treated cells were also left unstimulated (−) or co-stimulated (+, MG132). An equal number of cells were lysed for each sample. Cell lysates were immunoprecipitated using anti-GFP antibody and immunoblotted for ubiquitin. Similar amounts of WAVE2 were precipitated for each sample by using the precipitating antibody as the limiting factor. Ubiquitylated YFP-WAVE2 appears as a smear of bands around 110 kDa. Data shown are representative of four independent experiments. (**b**) Jurkat T cells were left unstimulated (−), or were co-stimulated with anti-CD3 and anti-CD28 antibodies (+). Cell lysates were immunoprecipitated using anti-Ub antibody and immunoblotted for WAVE2. Ubiquitylated endogenous WAVE2 appears as a smear of bands above the MW of 75 kDa with a prominent band around 83 kDa. Data shown are representative of three independent experiments. (**c**) Upper panel: Human primary peripheral-blood lymphocytes (PBLs) were left unstimulated (−) or were co-stimulated with anti-CD3 and anti-CD28 antibodies (+). MG132-treated cells were also left unstimulated (−) or co-stimulated (+, MG132). An equal number of cells were lysed for each sample. Cell lysates were immunoprecipitated using anti-WAVE2 antibody and immunoblotted for ubiquitin. Similar amounts of WAVE2 were precipitated for each sample by using the precipitating antibody as the limiting factor. Ubiquitylated endogenous WAVE2 appears as a smear of bands above the MW of 75 kDa, with a prominent band at ~83 kDa. Data shown are representative of at least three independent experiments. Lower panel: Cell lysates were prepared from activated and non-activated PBLs that were either pretreated with MG132 or left untreated, and analyzed for WAVE2 protein levels. WAVE2 expression levels were measured by ImageJ. Densitometry results for WAVE2 after normalization relative to the GAPDH values are presented.

We recently demonstrated that WAVE2 is recruited to the TCR site and co-localizes with the nucleation promoting factor (NPF), WASp, during the initiation of T-cell activation^15^. Previously, we showed that following recruitment to TCR-based clusters, WASp undergoes ubiquitylation-dependent degradation^34^. This mode of downregulation raised the question of whether activated WAVE2 molecules might undergo a similar process. To identify such a pathway, we examined whether this protein is ubiquitylated in T cells. We also determined whether this ubiquitylation is proteasome-mediated and dependent upon TCR stimulation. Untreated Jurkat T cells or MG132 proteasome inhibitor-pretreated cells were either left unstimulated, or were co-stimulated with anti-CD3 and anti-CD28 antibodies. Following TCR stimulation, immunoprecipitation of YFP-WAVE2 resulted in the co-precipitation of ubiquitylated WAVE2. The ubiquitylation of WAVE2 appeared as a smear of bands above the molecular weight (MW) of YFP-WAVE2 (~102 kDa) with a prominent band around 110 kDa, in a pattern similar to the other ubiquitylated proteins in T cells, WASp^34^, and Nck^35^ (Fig. 1a). This ubiquitylation was increased upon TCR stimulation. Furthermore, inhibition of the proteasome using MG132 led to an accumulation of ubiquitylated YFP-WAVE2 proteins, indicating that WAVE2 degradation is proteasome-dependent (Fig. 1a). These results were confirmed by a reciprocal immunoprecipitation, demonstrating a ladder of bands of ubiquitylated endogenous WAVE2 above the MW of WAVE2 (75 kDa) with a strong precipitate of ubiquitylated WAVE2 at around 83 kDa, which is increased in stimulated T cells (Fig. 1b). Also, ubiquitylation of WAVE2 was detected in non-transformed, human primary peripheral-blood lymphocytes (PBLs), and appeared as a smear of bands above the MW of endogenous WAVE2 (75 kDa) with a prominent band around 83 kDa (Fig. 1c, upper panel). This ubiquitylation was further enhanced by the presence of MG132. In addition, as indicated in the whole-cell lysates (WCL) prepared from these samples, MG132 treatment led to the accumulation of WAVE2 in both stimulated and unstimulated cells (Fig. 1c, lower panel). WAVE2 appears as two adjacent bands. As serine 351 residue accounts for the majority of ERK-induced WAVE2 phosphorylation^36^, we validated the upper MW, phosphorylated form of WAVE2 by immunoblotting with a phospho-specific (Ser(P)-351) WAVE2 antibody (see Supplementary Fig. S1).

To confirm that the ubiquitylated band detected by WAVE2 immunoprecipitation consisted of modified WAVE2 and not WAVE2-associated proteins, we immunoprecipitated WAVE2 from PBLs and boiled the immunoprecipitated proteins in SDS, allowing them to disassociate, and then re-immunoprecipitated WAVE2. As shown in Supplementary Fig. S2, a ubiquitylated band at ~83 kDa was detected in both the first and the second WAVE2 immunoprecipitations, indicating that the band detected by the Ub antibody represents directly ubiquitylated WAVE2. As expected, WAVE2-Hem1 co-immunoprecipitate was demonstrated only in the first two lanes and not in re-immunoprecipated lanes (third and fourth lanes).

### Mapping of the WAVE2 ubiquitylation site

**Figure 2:**
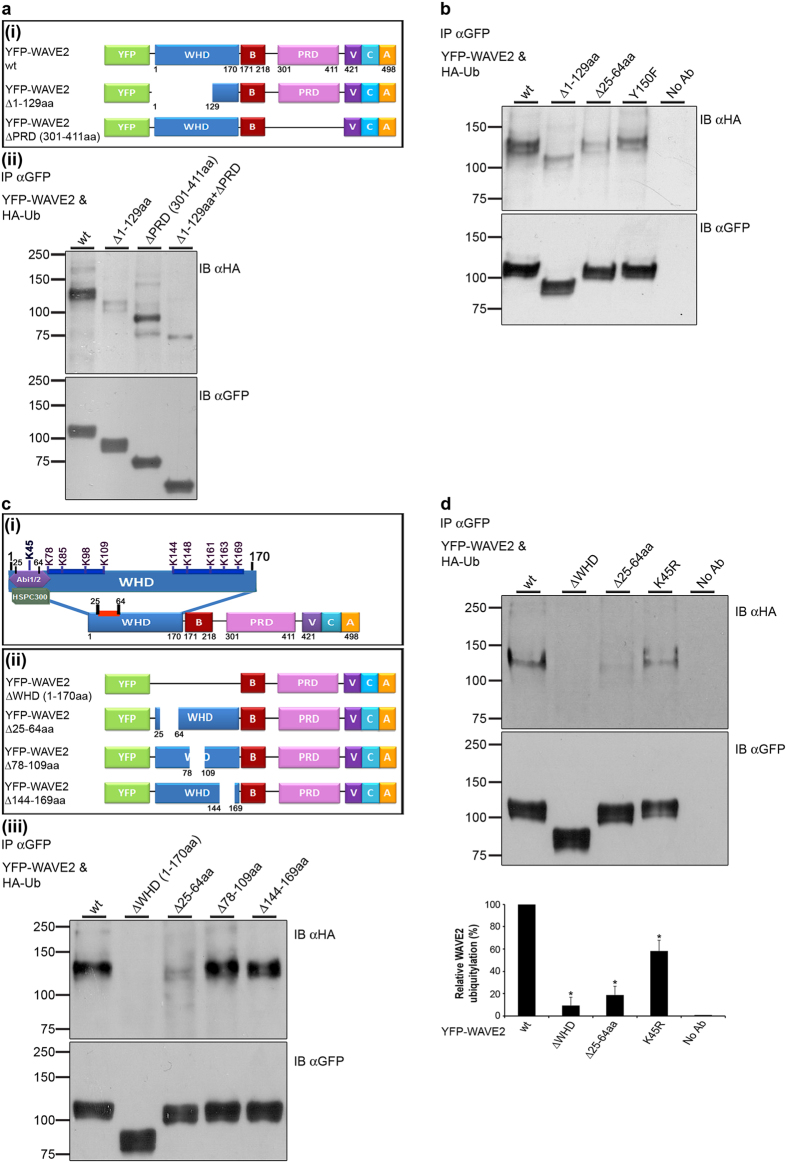
Identification of WAVE2 domains and lysine residues required for its ubiquitylation. (**a**(i)) Schemes of YFP-WAVE2 wt and YFP-WAVE2 deletion mutants. **WHD**, WAVE homology domain; **B**, basic region; **PRD**, proline-rich domain; **VCA**, verprolin-homology cofilin-homology acidic domain. (**a(**ii)) 293 T cells were co-transfected with constructs encoding HA-tagged ubiquitin together with YFP-WAVE2 wt or with YFP-WAVE2 mutant forms as indicated. YFP-WAVE2 was immunoprecipitated using anti-GFP antibody; complexes were resolved by SDS-PAGE and immunoblotted for ubiquitin using anti-HA antibody, and for YFP-WAVE2 using anti-GFP antibody. Ubiquitylated YFP-WAVE2 wt appears at a MW of ~110 kDa, which is 8 kDa above the MW of YFP-WAVE2 (~102 kDa). Data shown are representative of at least three independent experiments. (**b**) 293 T cells were co-transfected with constructs encoding HA-tagged ubiquitin together with YFP-WAVE2 wt or with YFP-WAVE2 mutant forms as indicated. Cells were lysed and immunoprecipitated with anti-GFP. IPs were separated by SDS-PAGE, transferred, and blotted with anti-HA and anti-GFP antibodies. Data shown are representative of three independent experiments. (**c**(i)) Schematic illustration of WAVE2 structural domains. The WAVE2 WHD domain contains ten lysine residues, as indicated. (**c**(ii)) Schemes of YFP-WAVE2 deletion mutants. (**c**(iii)) 293 T cells were co-transfected with constructs encoding HA-tagged ubiquitin together with YFP-WAVE2 wt or with YFP-WAVE2 mutant forms described in cii. Cells were lysed and immunoprecipitated with anti-GFP. IPs were separated by SDS-PAGE, transferred, and blotted with anti-HA and anti-GFP antibodies. Data shown are representative of at least four independent experiments. (**d**) 293 T cells were co-transfected, immunoprecipitated, and blotted as described above. Densitometric analysis of the bands presented was performed using ImageJ and normalized to the GFP densitometry values. Error bars show standard error of the mean (SEM). Significance was determined by two-tailed Student’s t-test and is indicated by asterisks (*P < 0.05). Data shown are representative of at least three independent experiments.

To identify the ubiquitylation site on WAVE2, we expressed a series of YFP-tagged deleted mutants of WAVE2 in 293 T cells, together with HA-Ub, and assessed their ubiquitylation. Deletion of the first 129aa of the WHD domain of WAVE2, (YFP-WAVE2 Δ1-129aa, Fig. 2a(i)), but not of amino acids 144–169 which are located at the end of this domain (YFP-WAVE2 Δ144-169aa), caused a dramatic decrease in WAVE2 ubiquitylation, compared to WAVE2 wt (Fig. 2a(ii) and Supplementary Fig. S3). Deletion of the PRD Δ301-411aa (YFP-WAVE2 ΔPRD, Fig. 2a(i)) had no effect on the extent of WAVE2 ubiquitylation (Fig. 2a(ii)). However, it should be noted that deletion of the PRD alone or in combination with the WHD, caused a complete loss of one of the two bands seen in all other mutant forms. Since ERK phosphorylates WAVE2 within its PRD^36^, it is possible that deletion of this site abrogates ERK phosphorylation, as detected by the exclusive appearance of the lower MW, unphosphorylated form of WAVE2.

A previous study showed that Abl-mediated phosphorylation of WAVE2 on tyrosine 150, located within the WHD, induces its activation and subsequent WAVE2-driven actin polymerization^32^. To further elucidate the association between WAVE2 activation and its ubiquitylation, we generated a YFP-WAVE2 construct containing a phospho-abolishing mutation on tyrosine 150 (YFP-WAVE2 Y150F). We then examined how the expression of this mutant form affects WAVE2 ubiquitylation. As seen in Fig. 2b, the phospho-abolishing mutation reduced WAVE2 ubiquitylation by more than 1.9 fold in comparison to WAVE2 wt (compare lane 1 to lane 4) indicating that the phosphorylated, active WAVE2 undergoes ubiquitylation, thereby supporting the role of ubiquitylation as a mechanism of negative WAVE2 regulation. Altogether, these data indicate that the WHD domain of WAVE2 mediates its ubiquitylation. Based on these results, we further dissected the function of this domain.

As shown in Fig. 2c(i), WHD contains ten lysine residues. Based on the location of these residues, we divided the WHD into subdomains, and prepared additional YFP-WAVE2 deleted mutant forms (Fig. 2c(ii)). The mutants prepared included WAVE2 ΔWHD; WAVE2 Δ25-64aa, which contains one lysine, and was previously found to serve as a docking site for the regulatory complex^30^; WAVE2 Δ78-109aa, which includes four lysine residues; and WAVE2 Δ144-169aa, which includes five lysine residues. YFP-WAVE2 mutant forms were expressed and tested for ubiquitylation by immunoprecipitation of WAVE2, and IB with anti-HA. Complete deletion of the WHD (YFP-WAVE2 ΔWHD 1-170aa) entirely abolished WAVE2 ubiquitylation, compared to the wt form (Fig. 2c(iii)), while no reduction in WAVE2 ubiquitylation was detected following deletion of amino acids 78–109, or amino acids 144–169, compared to WAVE2 wt (Fig. 2c(iii), compare lanes 4 and 5 to lane 1). Interestingly, deletion of amino acids 25–64 led to a substantial reduction in WAVE2 ubiquitylation (Fig. 2c(iii), compare lanes 3 and 1). This decrease upon deletion of the WRC binding site (Δ25-64aa) could be explained by the absence of lysine 45, which is the only lysine residue located within this region, or may be due to the lack of association with the other members of theWAVE2 complex that might be required for WAVE2 ubiquitylation. To test whether WAVE2 is ubiquitylated on lysine 45, we specifically mutated this lysine (YFP-WAVE2 K45R). Immunoprecepitation of the YFP-WAVE2 K45R mutant exhibited reduced ubiquitylation compared to wt WAVE2 (*P* ≤ 0.04) (Fig. 2d, lanes 4 vs. 1), although the decrease was less pronounced than that obtained by removing the WHD or the WRC binding site (*P* ≤ 0.006, and *P* ≤ 0.009, respectively) (Fig. 2d). These data indicate that WAVE2 is ubiquitylated on lysine 45, and suggest the involvement of members of the complex in the ubiquitylation and degradation process. To eliminate the possibility that the K45R mutation impairs the binding of WAVE2 to the WRC, we immunoprecipitated three forms of WAVE2 (wt, K45R, and Δ25-64aa) and compared their binding to the WRC protein, Hem-1. As expected, while the deletion of the WRC binding site disrupted this interaction, the K45R mutation did not affect the WAVE2-Hem-1 interaction (see Supplementary Fig. S4).

### The role of the WRC in WAVE2 ubiquitylation

**Figure 3:**
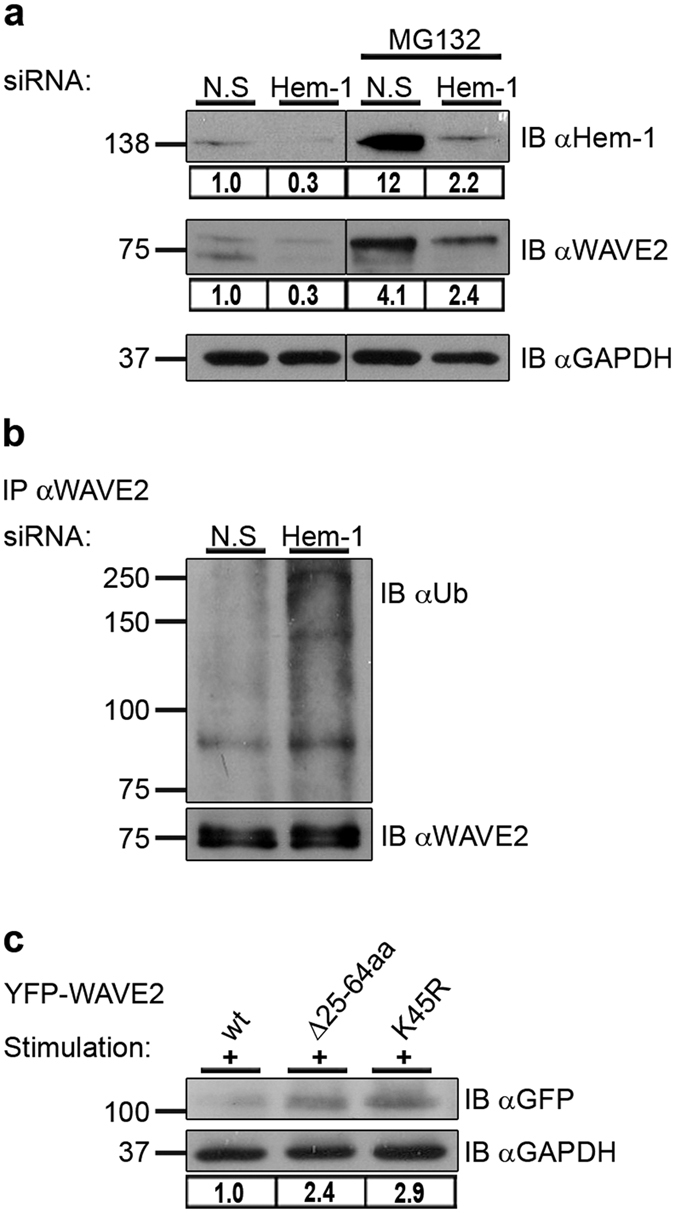
The role of the WRC in WAVE2 ubiquitylation. (**a**) Jurkat T cells were transfected with siRNA specific to human Hem-1 as well as with a non-specific (N.S) scrambled siRNA control. After 24 h, cells were treated with MG132 or left untreated, followed by co-stimulation with anti-CD3 and anti-CD28 antibodies. Cell lysates were prepared and analyzed for Hem-1 and WAVE2 protein levels. The gene-silencing efficiencies of Hem-1 as well as WAVE2 expression levels were measured by ImageJ. Densitometry results for Hem-1 or WAVE2 after normalization relative to the GAPDH values are presented. Vertical lines indicate noncontiguous blots. All lanes are from the same film and exposure time. (**b**) Lysates described in (**a**) from cells that were not treated with MG132, were immunoprecipitated using anti-WAVE2 antibody and immunoblotted for ubiquitin. Ubiquitylated endogenous WAVE2 appears above the MW of 75 kDa with a prominent band at ~83 kDa. (**c**) Jurkat T cells expressing YFP-WAVE2 wt or the YFP-WAVE2 mutant forms, Δ25-64aa and K45R, were sorted for YFP-positive cells. Cells were co-stimulated with anti-CD3 and anti-CD28 antibodies followed by lysis. Lysates were analyzed for WAVE2 protein levels by immunoblotting with anti-GFP antibody or anti-GAPDH as a loading control. Densitometric analysis of the bands presented was performed using ImageJ and normalized to the GAPDH densitometry values. Data shown are representative of three independent experiments.

To further determine the role of the complex components in WAVE2 degradation, the hematopoietic specific member, Hem-1, was gene silenced in T-cells using specific siRNA. A control of cells treated with a non-specific (N.S) scrambled siRNA pool served as a negative control. The cells were then treated with MG132 or left untreated followed by stimulation. Hem-1 expression was reduced by 70%, as analyzed by densitometry (Fig. 3a). Interestingly, gene silencing of Hem-1 caused a substantial decrease in WAVE2 expression. To verify that the resulting decrease in WAVE2 expression was caused by the silencing of Hem-1 rather than by an off-target effect of the siRNA used, we performed an experiment in which a different Hem-1-specific siRNA was used. A similar reduction in WAVE2 level was observed, demonstrating that the effect was specific to Hem-1 gene silencing (see Supplementary Fig. S5). In the presence of MG132 (Fig. 3a, right panel), a substantial accumulation of Hem-1 and WAVE2 was demonstrated in both N.S and, to a lesser extent, in Hem-1 siRNA-treated cells (Fig. 3a). Silencing of Hem-1 further enhanced WAVE2 ubiquitylation as determined by immunoprecipitation analysis of activated T cells, indicating that the presence of Hem-1 is required for WAVE2 stability (Fig. 3b). Furthermore, since knockdown of Hem-1 led to an increase in WAVE2 ubiquitylation and its consequent degradation, it is likely that the WRC binding site (amino acids 25-64) contains a degradation site for WAVE2.

To further examine the importance of the WRC binding site for WAVE2 stability, we used T cells stably expressing YFP-WAVE2 wt, or its mutant forms, Δ25-64aa or K45R. Lysates were prepared from YFP sorted cells (see Supplementary Fig. S6). A substantial accumulation of the WAVE2 mutant forms, i.e., WAVE2 Δ25-64aa and WAVE2 K45R was seen relative to WAVE2 wt following activation; protein levels of these mutant forms were increased by 2.4 and 2.9 fold, respectively (Fig. 3c), indicating that the 25–64 aa fragment and specifically lysine 45 play a role in activation-dependent WAVE2 degradation.

### The VCA domain plays a crucial role in WAVE2 stability

**Figure 4:**
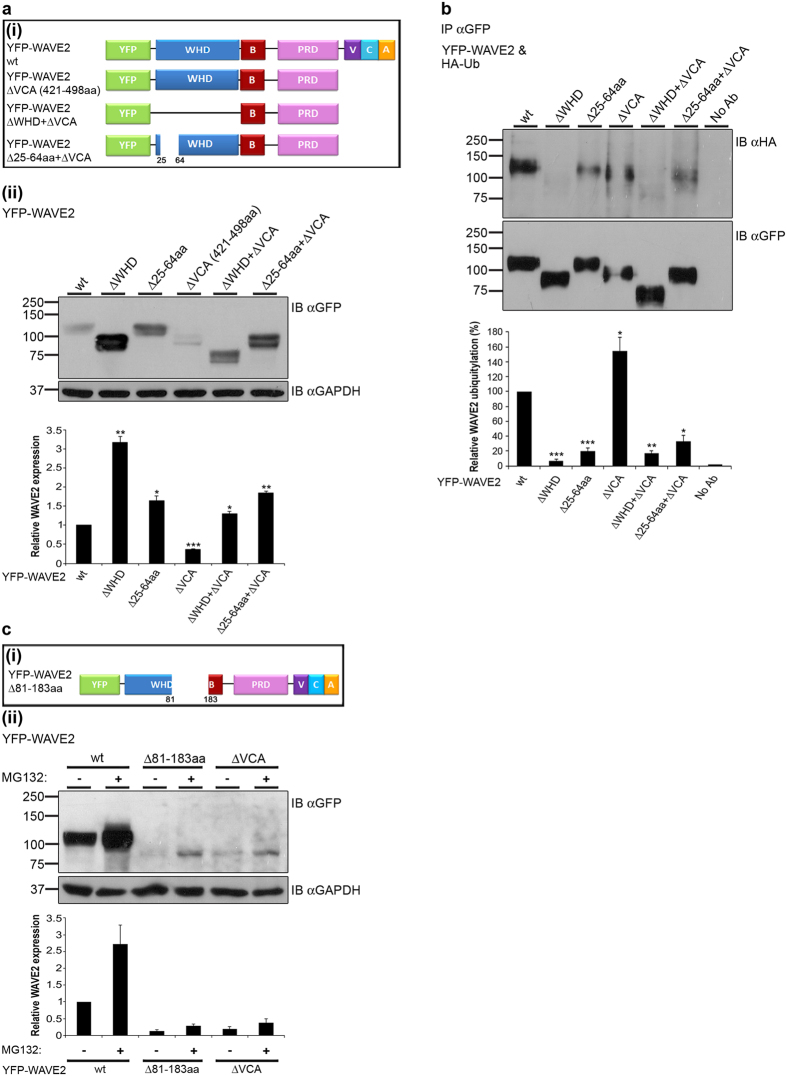
The VCA domain is required for WAVE2 stability. (**a**(i)) Schemes of YFP-WAVE2 deletion mutants. (**a**(ii)) 293 T cells were transfected with constructs encoding YFP-WAVE2 wt or YFP-WAVE2 mutant forms as indicated. Cells were lysed and analyzed for WAVE2 protein levels by immunoblotting with anti-GFP antibody or anti-GAPDH as a loading control. Densitometric analysis of the bands presented was performed using ImageJ and normalized to the GAPDH densitometry values. Data shown are representative of (blot), or averages of (lower graph) five independent experiments. Error bars show SEM. Significance was determined by two-tailed Student’s t-test and is indicated by asterisks (*P < 0.05; **P < 0.005; ***P < 0.0005). (**b**) 293 T cells were co-transfected with constructs encoding HA-tagged ubiquitin together with YFP-WAVE2 wt or with YFP-WAVE2 mutant forms, as indicated. YFP-WAVE2 was immunoprecipitated by anti-GFP, and the membranes were blotted with anti-HA and anti-GFP. Ubiquitylated YFP-WAVE2 wt appears at a MW of ~110 kDa. Densitometric analysis of the bands presented was performed using ImageJ and normalized to the GFP densitometry values. Data shown are representative of (blot), or averages of (lower graph) three independent experiments. Error bars show SEM. Significance was determined by two-tailed Student’s t-test, and is indicated by asterisks (*P < 0.05; **P < 0.005; ***P < 0.0005). (**c**(i)) Scheme of 81-183aa-deleted YFP-WAVE2. (**c**(ii)) 293 T cells were transfected with constructs encoding YFP-WAVE2 wt or YFP-WAVE2 mutant forms, as indicated. Cells were left untreated or pretreated with 0.5 μM MG132 overnight, lysed, and analyzed by immunoblotting, as described above. Densitometric analysis of the bands was performed using ImageJ and normalized to the GAPDH densitometry values.

Next, we examined whether the VCA domain of WAVE2 plays a role in WAVE2 stability in addition to the WRC binding site (fragment 25-64aa). Thus, several additional YFP-WAVE2 mutant forms were prepared, including WAVE2 ΔVCA, WAVE2 ΔWHD + ΔVCA, and WAVE2 Δ25-64aa + ΔVCA (Fig. 4a(i)). YFP-WAVE2 mutant forms were introduced into 293 T cells, and analyzed for their expression by western blot analysis. As shown in Fig. 4a(ii), deletion of the VCA domain caused a significant decrease (2.7 ± 0.01 fold) in WAVE2 protein levels compared to wt (*P* ≤ 0.0003), indicating that the VCA domain is crucial for maintaining WAVE2 stability. Not surprisingly, deletion of the WHD or the WRC binding site alone (Δ25-64aa), led to a remarkable accumulation of these non-ubiquitylated forms in comparison to WAVE2 wt (Fig. 4a(ii)). Quantification of protein expression indicated that YFP-WAVE2 ΔWHD or Δ25-64aa protein levels were increased by more than 3.1 ± 0.1 and 1.6 ± 0.1 fold, respectively, as compared to cells expressing wt WAVE2 (*P* ≤ 0.005, and *P* ≤ 0.03, respectively) (Fig. 4a(ii)). Deletion of the VCA domain together with the WHD or the WRC binding site restored WAVE2 protein expression to higher levels by more than 1.3 ± 0.05 and 1.8 ± 0.04 fold, respectively, as compared to full length WAVE2 wt (*P* ≤ 0.02, and *P* ≤ 0.002, respectively) (Fig. 4a(ii)).

Next, we examined the ubiquitylation of these mutant forms. WAVE2 deletion constructs of the WHD, 25-64aa, and VCA domains, alone or in combination, were co-expressed with HA-Ub followed by GFP immunoprecipitation and anti-HA immunoblotting. As previously shown, deletion of the WHD completely abolished WAVE2 ubiquitylation, while deletion of the WRC binding site caused a substantial decrease in WAVE2 ubiquitylation (Fig. 4b). Deletion of the VCA domain, on the other hand, resulted in increased ubiquitylation of WAVE2, suggesting that the VCA domain is involved in WAVE2 stability (Fig. 4b and Supplementary Fig. S7). As expected, expression of the WAVE2 ΔWHD + ΔVCA mutant showed no ubiquitylation, while expression of the WAVE2 Δ25-64aa + ΔVCA showed a substantial reduction in the ubiquitylation (Fig. 4b).

To further establish the effect of the VCA domain on WAVE2 stability, 293 T cells expressing YFP-WAVE2 wt, Δ81-183aa (which serves as the VCA binding site within WAVE2, Fig. 4c(i))^28^, or ΔVCA were pretreated with MG132, or left untreated. Cells were then analyzed for YFP-WAVE2 expression. Deletion of amino acids 81-183 or the VCA domain resulted in a substantial reduction in WAVE2 protein levels by more than 2 fold compared to the wt form (Fig. 4c(ii), compare lanes 3 or 5, respectively, to lane 1). Interestingly, WAVE2 protein levels were markedly increased in cells expressing WAVE2 Δ81-183aa or WAVE2 ΔVCA that were pretreated with 0.5 μM MG132 overnight, although to a lesser extent than YFP-WAVE2 wt (Fig. 4c(ii)), indicating a rapid and intense degradation enabled by removal of amino acids 81-183 or the VCA domain. In order to confirm that the degradation process is mediated mainly by the proteasome-dependent pathway, we pretreated ΔVCA and wt WAVE2 expressing cells with 50 μM MG132 for 3 h. This indeed resulted in the complete rescue of the mutated WAVE2 (see Supplementary Fig. S8a). We additionally confirmed these findings using a cycloheximide chase assay to demonstrate that the proteasome-dependent process is the dominant WAVE2 degradation pathway, and to compare the stability of the WAVE ΔVCA mutant to that of the wt (see Supplementary Fig. S8b,c). To validate that the VCA domain is essential for WAVE2 stability in T cells, we transfected Jurkat T cells with either YFP-WAVE2 wt or YFP-WAVE2 ΔVCA, and demonstrated reduced expression of the mutant via western and FACS analyses (see Supplementary Fig. S9). Taken together, our results suggest the importance of the VCA domain for WAVE2 stability.

### WAVE2 is found in an autoinhibitory conformation which is released following TCR activation

**Figure 5:**
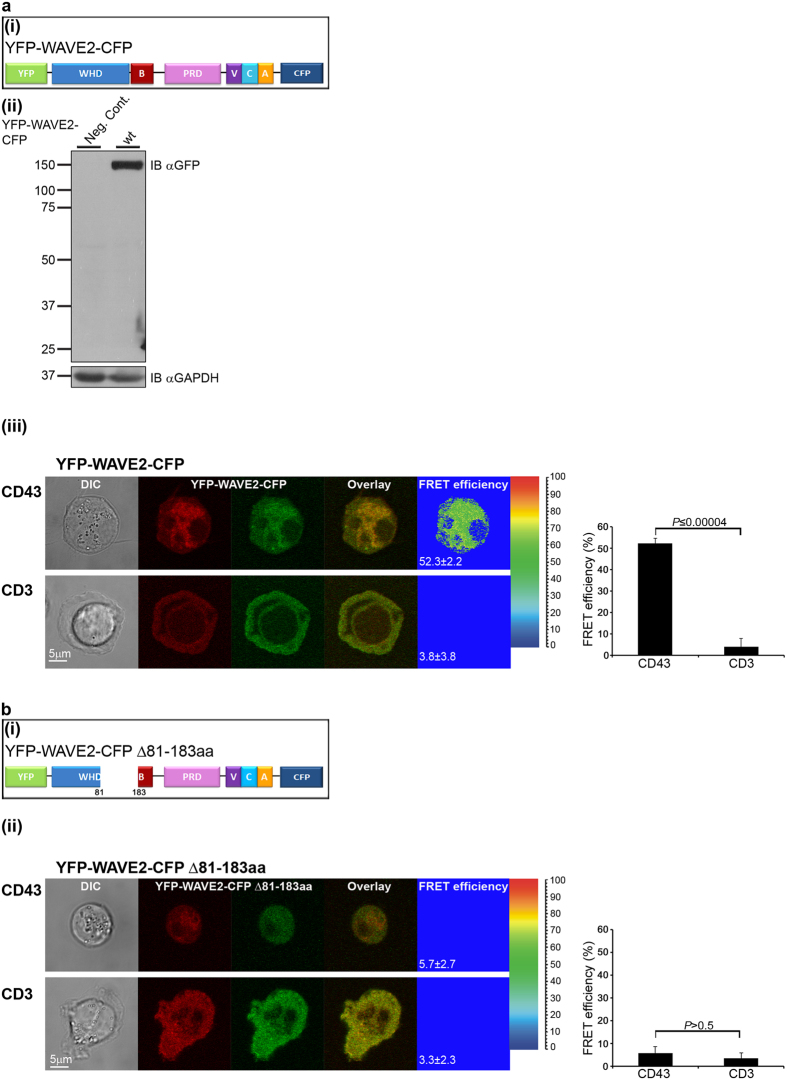
WAVE2 is released from an autoinhibitory conformation following TCR engagement. (**a**(i)) Schematic representation of the tagged WAVE2 protein used for FRET analysis. (**a**(ii)) Jurkat cells expressing YFP-WAVE2-CFP and untransfected cells (negative control – Neg. Cont.) were analyzed by western blotting with anti-GFP. GAPDH served as a loading control. (**a**(iii)) Jurkat T cells expressing YFP-WAVE2-CFP were plated over either non-stimulatory coverslips coated with anti-CD43 antibody (upper panel, n = 89 cells), or over stimulatory coverslips coated with anti-CD3 antibody (lower panel, n = 42 cells). Cells were fixed following 3 min of activation and imaged by confocal microscopy. FRET analysis was performed using the donor-sensitized acceptor emission technology (see Methods for details). A graph summarizing the mean FRET efficiency in cells expressing YFP-WAVE2-CFP plated over the indicated coverslips is presented on the right. Graph shows means ± SEM from three independent experiments. Significance was determined by two-tailed Student’s t-test. (**b**(i)) Scheme of 81-183aa-deleted YFP-WAVE2-CFP. (**b**(ii)) Jurkat T cells transfected with a plasmid encoding YFP-WAVE2-CFP Δ81-183aa were plated over either non-stimulatory (anti-CD43, upper panel, n = 76 cells) or stimulatory (anti-CD3, lower panel, n = 48 cells) coverslips and fixed after 3 min of activation. A graph showing the mean FRET efficiency in cells expressing YFP-WAVE2-CFP Δ81-183aa plated over the indicated coverslips is presented on the right. Graph shows means ± SEM from three independent experiments. Significance was determined by two-tailed Student’s t-test.

We then determined whether WAVE2 undergoes a conformational change that releases its VCA domain following TCR activation. Förster resonance energy transfer (FRET) technology enables the detection of intra- as well as inter- molecular interactions on a nanometer scale^37^,^38^. We utilized this technique to monitor possible conformational changes within WAVE2 by constructing a FRET-based probe consisting of WAVE2 tagged with YFP at its N-terminus and with CFP at its C-terminus (YFP-WAVE2-CFP, Fig. 5a(i)). To verify the cellular functionality of the probe, we investigated its tyrosine residue phosphorylation, which was previously shown to be critical for its activity^32^. Indeed, phosphorylation of YFP-WAVE2-CFP was found to be normal (see Supplementary Fig. S10a). Furthermore, we determined the cellular distribution of YFP-WAVE2-CFP in comparison to the previously described YFP-WAVE2 protein^15^ (see Supplementary Fig. S10b). Most importantly, we determined the ability of YFP-WAVE2-CFP to restore T cell activation-dependent LFA-1 conformational change in endogenous WAVE2 knockdown T cells. To this end, the YFP-WAVE2-CFP plasmid contains silent mutations that render it resistant to the siRNA used (see Methods). As was previously shown by us, WAVE2 knockdown disrupts the activation of LFA-1, as demonstrated using KIM127, an antibody specific to the activated conformation of LFA-1^15^. Here, we show that YFP-WAVE2-CFP expression rescues LFA-1 activation in WAVE2 knockdown cells, demonstrating the normal functionality of YFP-WAVE2-CFP (see Supplementary Fig. S10c).

T cells stably expressing YFP-WAVE2-CFP were plated over either non-stimulatory (anti-CD43) (Fig. 5a(iii), upper panel), or stimulatory (anti-CD3) (Fig. 5a(iii), lower panel) pre-coated coverslips, and fixed after 3 min of activation. The intra-molecular proximities of the doubly-tagged WAVE2 were measured by FRET analysis. High FRET efficiency of 52.3 ± 2.2% was measured in naïve cells, indicating a close proximity between the N-terminus of WAVE2 and the C-terminus. In contrast, the FRET efficiency measured between the N and the C- termini in stimulated T cells, was significantly reduced (3.8 ± 3.8%, *P* ≤ 0.00004), indicating that these regions were no longer in close proximity (Fig. 5a(iii) right graph). These data provide direct and compelling evidence that WAVE2 assumes an autoinhibitory conformation in its resting state, which is altered upon TCR activation.

We next established the necessity of residues 81-183 for the WAVE2 autoinhibitory conformation. We expressed the FRET-labelled WAVE2 probe with a deletion of residues 81–183 (YFP-WAVE2-CFP Δ81-183aa, Fig. 5b(i)) in T cells, and measured the FRET efficiency between CFP and YFP. As removing amino acids 81-183 led to massive degradation (Fig. 4c(ii)), transfected T cells were pretreated with MG132. Almost no FRET was measured in naïve (Fig. 5b(ii), upper panel) or activated cells (Fig. 5b(ii), lower panel) (5.7 ± 2.7% and 3.3 ± 2.3%, respectively, *P* > 0.5) (Fig. 5b(ii) right graph), indicating that deletion of the VCA binding site within the WAVE2 N-terminus, disrupts the WAVE2 autoinhibitory conformation. Overall, the FRET data clearly show that WAVE2 is found in an autoinhibitory conformation facilitated by intra-molecular interactions between its WHD and VCA domains. Disruption of these intra-molecular interactions by either TCR activation or deletion of the VCA binding site, results in a conformational change within the molecule, assuming a non-inhibited state (see schematic model in Supplementary Fig. S11a–c).

### WAVE2 conformational change dictates its ubiquitylation and consequent degradation

**Figure 6:**
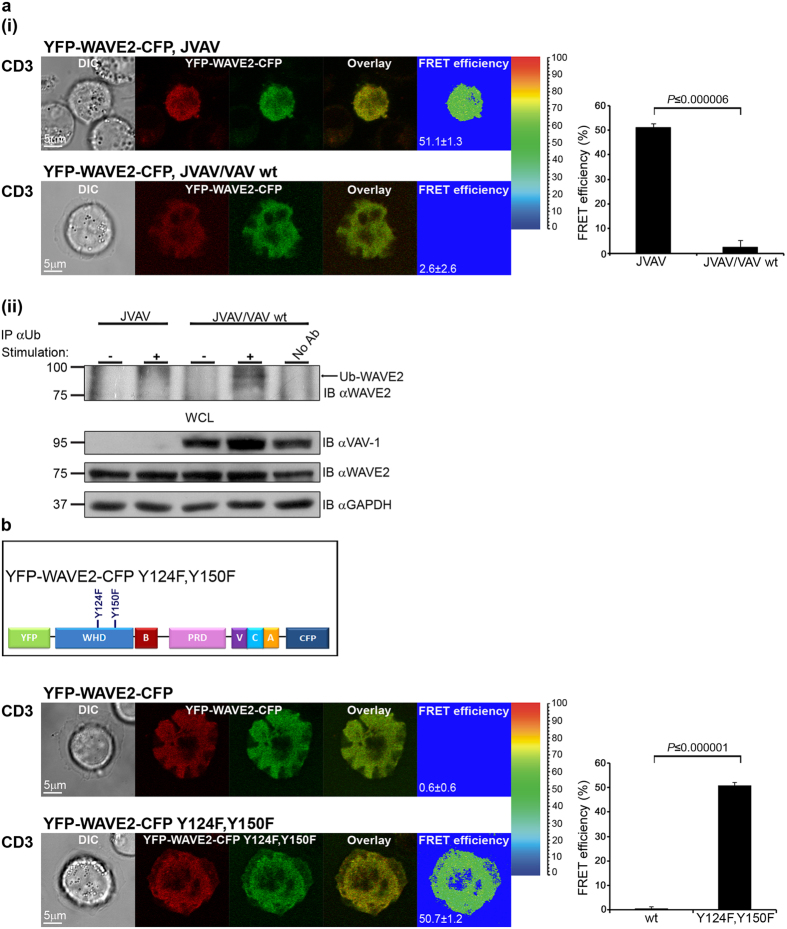
The open non-autoinhibited conformation of WAVE2 is ubiquitylated. (**a**(i)) Jurkat T cells deficient in VAV-1 (JVAV, upper panel, n = 10 cells), and JVAV cells reconstituted with VAV-1 wt (JVAV/VAV wt, lower panel, n = 16 cells) transfected with a plasmid encoding YFP-WAVE2-CFP were plated over stimulatory coverslips, fixed following 3 min activation, and the FRET efficiency between CFP and YFP was measured. A graph showing the mean FRET efficiency in the indicated cell types expressing YFP-WAVE2-CFP plated over stimulatory coverslips is presented on the right. Graph shows means ± SEM from three independent experiments. Significance was determined by two-tailed Student’s t-test. (**a**(ii)) JVAV and JVAV/VAV wt were left unstimulated (−), or were co-stimulated (+) with anti-CD3 and anti-CD28 antibodies. Cell lysates were subjected to immunoprecipitation (IP) with anti-Ub antibody, and samples were analyzed by western blotting (IB) with anti-WAVE2 antibody. Ubiquitylated WAVE2 appears at a molecular weight of 83 kDa. Bottom: WCLs before immunoprecipitation were analyzed by western blotting with the indicated antibodies. All western blots are representative of three independent experiments. (**b**) Scheme of point-mutated YFP-WAVE2-CFP with tyrosine 124 and 150 residues replaced by phenylalanine residues. Jurkat T cells transfected with plasmids encoding YFP-WAVE2-CFP wt (upper panel, n = 43 cells) or YFP-WAVE2-CFP Y124F, Y150F (lower panel, n = 21 cells) were plated over stimulatory coverslips, fixed following 3 min activation, and the FRET efficiency between CFP and YFP was measured. A graph showing the mean FRET efficiency in cells expressing either YFP-WAVE2-CFP wt or YFP-WAVE2-CFP Y124F, Y150F that were plated over stimulatory coverslips is presented on the right. Graph shows means ± SEM from three independent experiments. Significance was determined by two-tailed Student’s t-test.

So far, we have shown that WAVE2 conformational change and ubiquitylation are each dependent on cellular activation. We next investigated whether there is a linkage between these two processes. There are several activators of the WRC, among them, the small GTPase Rac1, an essential player in cell motility and chemotaxis^39^. However, WAVE proteins lack the ability to bind directly to small GTPases, and several proteins have been suggested to mediate the interaction between Rac1 and WAVE, including Sra1^40^. To determine the effect of WAVE2 activation by the Rac1 signaling cascade on its conformational structure, we examined the role of VAV-1, which functions as a guanine nucleotide exchange factor (GEF) for Rac1^41^, in releasing WAVE2 from its autoinhibited conformation. Jurkat T cells deficient in VAV-1 (JVAV), and JVAV cells reconstituted with VAV-1 wt (JVAV/VAV wt), expressing YFP-WAVE2-CFP were plated over stimulatory pre-coated coverslips, fixed, and the FRET efficiency between CFP and YFP was measured (Fig. 6a(i)). The FRET efficiency in JVAV was significantly higher than in JVAV cells reconstituted with VAV-1 (51.1 ± 1.3% and 2.6 ± 2.6%, respectively, *P* ≤ 0.000006), demonstrating that in the absence of VAV-1, WAVE2 is maintained in an autoinhibited conformation (Fig. 6a(i) right graph). Next, we determined whether the open non-inhibited conformation of WAVE2 is necessary for its ubiquitylation. JVAV and JVAV reconstituted with VAV-1 wt cells were left either unstimulated or co-stimulated with anti-CD3 and anti-CD28 antibodies followed by ubiquitin immunoprecipitation. WAVE2 ubiquitylation was analyzed; the prominent band of ~83 kDa, which represents WAVE2 ubiquitylation, was almost completely absent in VAV-1-deficient T cells following TCR activation (Fig. 6a(ii)). These results support our observations that WAVE2 conformational change occurs following its activation (Fig. 5a(iii)), and prove the significance of WAVE2 conformational state for its ubiquitylation.

Phosphorylation of WAVE proteins is also essential for WRC activation^32^,^36^,^42^,^43^ (reviewed in ref. 44), although the mechanisms through which it works are largely unknown. Previous studies have shown that phosphorylation of WAVE1, specifically at tyrosine 125 and tyrosine 151 (equivalent to tyrosine 124 and tyrosine 150, respectively, in WAVE2), could activate this protein^28^. To address whether phosphorylation of WAVE2 on tyrosine residues 124 and 150 destabilizes its autoinhibited conformation, leading to release of the VCA domain and consequent ubiquitylation, we constructed a FRET-based probe with phospho-abolishing mutations at the tyrosine 124 and 150 residues (YFP-WAVE2-CFP Y124F, Y150F, Fig. 6b). T cells expressing this mutant form were plated over stimulatory coverslips, and the FRET efficiency between CFP and YFP was measured (Fig. 6b). FRET efficiency was markedly higher in cells expressing YFP-WAVE2-CFP Y124F, Y150F, and similar to that in unstimulated cells (Fig. 5a(iii), upper panel), in contrast to cells expressing the wt form, which showed almost no FRET (50.7 ± 1.2% and 0.6 ± 0.6%, respectively, *P* ≤ 0.000001) (Fig. 6b right graph). These results indicate that WAVE2 is autoinhibited in YFP-WAVE2-CFP Y124F, Y150F expressing cells. Indeed as mentioned above, the WAVE2 Y150F phospho-abolishing mutation was less susceptible to ubiquitylation in comparison to WAVE2 wt (Fig. 2b), further establishing the association between WAVE2 conformational change and its ubiquitylation.

## Discussion

As key regulators of the actin cytoskeleton, WAVE proteins serve as hubs, linking upstream signals to the activation of the Arp2/3 complex, thereby mediating the propagation of signaling cascades that lead to actin polymerization.

In cells, WAVE proteins are found as part of a large molecular weight protein complex, designated as the WRC, with four additional participating proteins: Sra1, Nap1, Abi1/2, and HSPC300, or their homologues^24^,^45^.

Most studies have suggested that the WRC members are involved in WAVE stabilization^8^,^12^,^46^. However, the precise nature of the molecular mechanism underlying this regulation, and its biological significance are still unknown. In the current study, we aimed to characterize the molecular mechanisms governing the degradation of WAVE2, and to provide a comprehensive view of its negative regulation.

We demonstrate that WAVE2 ubiquitylation is induced upon TCR activation, and is mediated by the proteasome. We showed that the WHD, and specifically lysine 45, located within the WRC binding site, are essential for WAVE2 ubiquitylation. These results are supported by a previous study showing that *Dictyostelium* cells expressing the WAVE homologue, SCAR, lacking its WRC binding site produce a stable protein both in wild type cells, and under genetic backgrounds missing different members of the complex^30^. Strikingly, we demonstrate here that deletion of WAVE2 WHD or the WRC binding site (Δ25-64aa), leads to a substantial accumulation of these proteins in cells. Expression of the 25-64aa deleted mutant causes a lesser decrease in WAVE2 ubiquitylation than that of the ΔWHD mutant. Interestingly, mutation of lysine 45 did not completely abolish WAVE2 ubiquitylation, suggesting that other potential lysine residue or ubiquitylation motif/s located at the WHD, are also involved in this process.

WAVE proteins have been suggested to be inactive within the WRC at resting state^29^,^31^,^47^, and the resolution of the crystal structure of WAVE1 and its regulatory complex was documented^28^. However, it should be noted that in the recombinant WAVE1 complex used in the crystallographic studies, the C-terminal proline-rich region, as well as the SH3 domain of Abi2, were genetically removed, and the large PRD of WAVE1 was replaced with an 18-residue linker to enable crystallization, and were thus not determined. These domains are highly conserved and physiologically important. Therefore, in this study, we used intact WAVE2 to explore the linkage between WAVE2 activation and its conformational state with its degradation in a more physiological system. For this purpose, we utilized a FRET-based sensor which enabled WAVE2 conformation to be monitored *in vivo*. Using this sensor, we detected a direct interaction between the C-terminus of WAVE2 and its N-terminus in resting cells. Specifically, this intra-molecular interaction involved residues 81-183, which bind the C-terminal VCA domain. This autoinhibited conformation appeared to be dynamic, as activation of WAVE2 following TCR stimulation destabilized the intra-molecular interaction, releasing its autoinhibition. Importantly, the TCR-induced WAVE2 conformational change was abolished under conditions that prevent the activation of the WRC, i.e., abrogating Rac1-mediated activation, or perturbing WAVE2 phosphorylation, indicating the importance of proper WAVE2 activation for its conformational change.

The mode of WAVE2 negative regulation revealed here seems to resemble that of WASp^34^. Similar to WAVE2, WASp is intrinsically inactive in naïve T cells and exists in an autoinhibited state in which its C-terminal VCA domain intramolecularly interacts with its GTPase-binding domain (GBD)^48^ and, intermolecularly, with WASp-interacting protein (WIP)^37^. Furthermore, in these cells, WASp strongly associates with WIP via amino acids 56–102 located within the WASp N-terminal WH1 domain^34^,^49^,^50^. Deletion of the WASp VCA domain disrupts its interaction with WIP, thereby exposing WASp to ubiquitylation and, subsequently, to degradation^37^. In a manner similar to WASp regulation, WAVE2 also requires the release from its autoinhibitory conformation by dissociating the VCA domain. Furthermore, we previously discovered that upon TCR stimulation, the WASp-WIP molecular complex is partially dissociated, leading to the ubiquitylation of activated WASp on lysine residues 76 and 81 and to its degradation^34^,^37^. Indeed, we show here the essential role of the non-inhibited open conformation of WAVE2 for its ubiquitylation. The prevention of WRC activation not only abolished WAVE2 conformational change, but also caused a substantial reduction in WAVE2 ubiquitylation, as demonstrated by biochemical analysis. Thus, a strong link was established between WAVE2 conformational change and its ubiquitylation. This activation-linked degradation mechanism is plausible for other structurally related proteins from the WASp family of proteins and requires further investigation.

Our data could be integrated to propose a novel mechanism of WAVE2 negative regulation. WAVE2 VCA domain serves as a cap which anchors the components of the WRC to their binding site on WAVE2 (25-64aa), thereby contributing to the masking and the protection of WAVE2 ubiquitylation site/s. Upon TCR engagement, WAVE2 is recruited to the TCR-APC contact site and associates with protein tyrosine kinases, i.e., ZAP-70 and the adaptor proteins LAT, SLP-76, VAV-1 and Nck, forming signaling clusters, which facilitate its activation and function^15^. WAVE2 activation then triggers a conformational change that releases the sequestered VCA domain, exposing WAVE2 WHD and, specifically, lysine 45, to ubiquitylation, promoting WAVE2 degradation (see Supplementary Fig. S11d).

WAVE2 deficiency is embryonic lethal, due to hemorrhages caused by aberrant actin rearrangement resulting in platelet destruction^7^. This indicates the crucial role of WAVE2 in actin dependent-cellular functions. The requirement of WAVE2 for the appropriate immune response is also suggested by studies of mice deficient or mutated in other members of the WRC. Accordingly, Hem-1 deficient mice, which exhibit lymphopenia, neutrophilia, and anemia, provide valuable hints at the possible physiological consequences of WAVE2 deficiency. T cells from Hem-1 deficient mice displayed impaired development, activation, and proliferation, caused by aberrant actin polymerization, and decreased integrin-mediated adhesion to fibronectin^51^. Since we show here that gene silencing of Hem-1 results in nearly complete degradation of WAVE2 (Fig. 3a,b), these defects could derive from the absence of WAVE2, which is heavily involved in these processes. Furthermore, it was demonstrated that in addition to integrin-mediated cellular adhesion and actin polymerization, WAVE2 is also essential for calcium flux, NFAT activation and IL-2 transcriptional activity^11^,^13^,^14^,^15^. Thus, enhancing the natural degradation cascade of WAVE2 might be an efficient way to prevent undesirable actin nucleation that may lead to hyper-activation of T cells as manifested in chronic inflammatory responses.

Hence, identification of the WAVE2 downregulation mechanism might provide a valuable framework for developing an innovative approach for regulating WAVE2-dependent processes in health and disease.

## Methods

### Antibodies and Reagents

Antibodies and their sources were as follows. Antibodies for imaging: Mouse anti-CD3ε (UCHT) and anti-CD43 (BD Biosciences). Primary antibodies for immunoprecipitation and immunoblotting: mouse anti-GFP, rat anti-HA peroxidase 3F10 (Roche Applied Science); mouse anti-GAPDH (Biodesign); rabbit anti-ubiquitin (Dako); rabbit anti-Hem-1 (Novus); goat anti-WAVE2 D16, mouse anti-ubiquitin P4D1, mouse anti-Vav D7 (Santa Cruz Biotechnology, Inc); rabbit anti-phospho WAVE2 (Ser-351), mouse anti-phosphotyrosine (pTy) 4G10 (Millipore). An additional WAVE2 antibody was kindly provided by D.D Billadeau from the College of Medicine, Mayo Clinic, MN, USA. Secondary antibodies for immunoblotting: goat anti-mouse (Sigma-Aldrich); goat anti-rabbit, donkey anti-goat (Santa Cruz Biotechnology, Inc). Alexa-conjugated, isotype-specific secondary antibody was purchased from Thermo Fisher Scientific. Pools of three independent RNA duplexes specific for human Hem-1 were obtained from Invitrogen, with the following sequences: CCACCUUCAGUACUUGGCAAGAUUU, CCAAGGUGAUGAACCUCAUUGUCUU, and CCUUGCCACUGACCCUUCUUCCUUU.

Additionally, another RNA duplex was obtained from Sigma-Aldrich, with the following sequence:

UCUAACAACAAGAACAUUGA.

An RNA duplex specific to endogenous human WAVE2 was obtained from Sigma-Aldrich, with the following sequence:

GAAGAGAAAGCACAGGAAA.

Pools of non-targeting (non-specific, N.S) negative control siRNA duplexes were purchased from Dharmacon and have the following sequences: UAGCGACUAAACACAUCAA, UAAGGCUAUGAAGAGAUAC, AUGUAUUGGCCUGUAUUAG, AUGAACGUGAAUUGCUCAA, and UGGUUUACAUGUCGACUAA.

### Expression vectors and plasmids

Silently mutated human WAVE2 cDNA was kindly provided by D.D. Billadeau from the College of Medicine, Mayo Clinic, MN, USA. These mutations render the product of this plasmid resistant to the WAVE2-specific siRNA used (targeted sequence was changed to aAAaAGgAAaCACAGGAAA; lowercase letters indicate a changed nucleotide that does not affect the amino acid sequence). The cDNA was cloned into pcDNA3 + Neomycin vector from Invitrogen, and then was cloned into the expression vectors pEYFP-C1 and pECFP-N1 from Clontech, to obtain the YFP or CFP-tagged WAVE2. GFP fusions were rendered monomeric by an A206K substitution described by Zacharias^52^. The YFP-WAVE2-CFP construct was created by insertion of the WAVE2-CFP segment, using restriction enzymes BamHI and NotI into a YFP-WAVE2 plasmid. WAVE2 point mutations, in which each lysine/tyrosine was substituted with arginine/phenylalanine, respectively, WAVE2 deleted mutants WAVE2 ΔWHD (1-170aa), WAVE2 Δ25-64aa, WAVE2 Δ78-109aa, WAVE2 Δ141-169aa, WAVE2 ΔPRD (301-419aa), WAVE2 ΔVCA (421-498aa), WAVE2 Δ81-183aa, and combinations of those deletions were introduced into the YFP-WAVE2/YFP-WAVE2-CFP constructs using Quickchange II XL site-directed mutagenesis kit (Stratagene) according to the manufacturer’s instructions. All constructs were verified using DNA sequencing. The HA-tagged ubiquitin plasmid was previously described^53^.

### Cell culture and transfection of 293 T cells

Human Embryonic Kidney 293 T cells were cultured in DMEM, supplemented with 10% FBS, 2 mM L-glutamine, 50 μg/ml penicillin, and 50 μg/ml streptomycin. The 293 T cells were transiently transfected by DNA-calcium phosphate co-precipitation, according to the manufacturer’s instructions (Biontex).

### Transfection of Jurkat T cells, generation of stable cells and FACS analysis

Jurkat E6.1 T cells were transfected with either a Lonza Nucleofector™ 2b Device using the manufacturer’s protocol H-10, or Bio-Rad electroporator settings: 1000 μF, 254 v. Transiently transfected T cell cultures were used in this study 48 h after transfection. Stable clones were derived from transiently transfected cells with a combination of drug selection and cell sorting. Cells transiently expressing chimeric proteins were selected in neomycin. Fluorescence analysis and cell sorting were performed using the FACSVantage (Becton Dickinson Biosciences) and FlowJo software^54^,^55^.

### PBL isolation and stimulation

Anonymized peripheral-blood lymphocytes (PBLs) were obtained from healthy donors through the blood services center of the Sheba Medical Center in accordance with the approved guidelines, and informed consent was obtained from all donors. All experimental protocols were approved by the Israeli National Blood Bank, and conducted according to approved guidelines from Magen David Adom in Israel, the Israel Red Cross National Society. PBLs were isolated by Ficoll density gradient centrifugation, as previously described^56^. The cells were activated with anti-CD3ε (OKT3, 10 μg/ml) and anti-CD28 (10 μg/ml) for 30 min on ice. The cells were then warmed to 37 °C for 10 min and stimulated with anti-human IgG (50 μg/ml) for 2 min.

### siRNA treatment

Cells were transfected with control siRNA or siRNA specific for human Hem-1 or endogenous human WAVE2 using a Lonza Nucleofector™ 2b device and the manufacturer’s protocol H-10.

### Immunoprecipitation and immunoblotting

T cells were either stimulated with anti-CD3ε (OKT3, 10 μg/ml) and anti-CD28 (10 μg/ml) antibodies for 2 min at 37 °C, or left untreated followed by lysis. PBLs were stimulated and lysed, as previously described^57^. Transfected 293 T cells were lysed 48 h after transfection in ice-cold NP-40 lysis buffer containing 50 mM Tris-HCl pH 7.4, 150 mM NaCl, 5 mM EDTA, 5 mM EGTA, 50 mM NaF, 1% NP-40 and complete protease inhibitor tablets (Roche Applied Science). Proteasome activity was blocked by addition of MG132 (Sigma-Aldrich) to PBLs and T- cell medium at a final concentration of 50 μM for 3 h before the cells were harvested. 293 T cells were incubated overnight with 0.5 μM MG132. Immunoprecipitation and immunoblotting were performed as previously described^57^. Denaturation of immunoprecipitated complexes and re-precipitation of the WAVE2 protein were performed as previously described^53^. Briefly, the precipitated complex was resuspended and boiled for 5 min in 100 μl of denaturation buffer (20 mM Tris-HCl pH 8, 50 mM NaCl, 5 mM DTT, 1% SDS, and 1 mM sodium orthovanadate), the denatured sample was then diluted to 1 ml with cell lysis buffer, and a second immunoprecipitation was performed as described above. Densitometric analysis was performed using ImageJ software with final results normalized using GAPDH or GFP as loading controls for whole-cell lysates and immunoprecipitated samples, respectively. Relative protein abundance or relative amount of co-precipitated protein was compared to the relevant control.

### Cycloheximide chase assay

WAVE2 (wt or ΔVCA) protein stability was determined in the presence or absence of the protein biosynthesis inhibitor, cycloheximide (Sigma-Aldrich), and in the presence or absence of proteasomal inhibitor MG132. 293 T cells were transfected with either YFP-WAVE2 wt or ΔVCA and were plated into 60-mm plates. After 24 h, fresh medium containing cycloheximide (0.1 mg/ml) was added. At the indicated time intervals (0 h, 2 h, 4 h, and 6 h) cells were lysed as previously described^57^. To assess the effect of MG132 on the turnover of WAVE2, cells were simultaneously treated with 50 μM MG132 and 0.1 mg/ml cycloheximide for the indicated time intervals.

### Confocal Microscopy: Spreading assay and FRET analysis

#### Spreading assay

Spreading assays were performed as previously described^56^,^58^. Briefly, T cells (2 × 10^6^ cells/ml) were seeded on the bottom of chambered cover glasses (Lab-Tek) that were precoated with anti-CD3 stimulatory monoclonal antibodies (10 μg/ml). The cells were incubated in imaging buffer (RPMI without phenol red containing 10% fetal calf serum and 25 mM Hepes) at 37 °C, 5% CO2, for 3 min. Cells were fixed for 25 min with 2.5% paraformaldehyde in phosphate-buffered saline (PBS) and then were washed three times with PBS. Dynamic fluorescent and differential interference contrast (DIC) images were collected on a Zeiss LSM510 Meta confocal microscope. All images were collected with a 63x Plan-Apochromat objective (Carl Zeiss).

#### FRET analysis

Förster resonance energy transfer (FRET) was measured by the donor-sensitized acceptor fluorescence technique. Three sets of filters were used: one optimized for donor fluorescence (excitation, 468 nm; emission, 475−505 nm); a second, for acceptor fluorescence (excitation, 514 nm; emission, 530 nm LP); and a third, for FRET (excitation, 468 nm; emission, 530 nm LP). FRET was corrected and its efficiency was determined as described in detail by us^15^,^37^,^38^,^58^.

### LFA-1 activation assay

The activation of LFA-1 integrins was determined by flow cytometry, as previously described^59^. Briefly, 3 × 10^5^ cells were stimulated with 5 μg/ml anti-CD3 at 37 °C for 15 min. After stimulation, cells were washed and incubated with the conformation-sensitive anti-LFA-1 KIM127 mAb at 37 °C for 30 min. Cells were then washed and incubated with Alexa 568-conjugated goat anti-mouse IgG1 on ice for 30 min. After washing, cells were analyzed with a FACSARIA III Flow Cytometer (Becton Dickinson Biosciences).

### Analysis of WAVE2 cellular distribution

Analyses of YFP-WAVE2-CFP and YFP-WAVE2 distribution along the diameter of the spreading cell were performed by measuring the fluorescence intensity along virtual lines stretching from one point at the cell’s periphery, through the center, to the other side of the cell. Two perpendicular lines were drawn and calculated for each imaged cell. Cell diameter was normalized to 1, and results from each sampled group were divided into 100 bins representing their relative location along the diameter of the synapse. For each bin, the average intensity was calculated and normalized using the average intensity of each measured line to determine WAVE2 fold intensity along the diameter. All YFP-WAVE2-CFP and YFP-WAVE2 distribution analyses were performed 3 min following activation and using ImageJ. PERL and Microsoft Excel were used for data binning and statistics.

### Statistical analyses

Standard errors were calculated with the use of Microsoft Excel. Student’s t test was used to evaluate significance. In all cases, the threshold *P* value required for significance was 0.05.

## Additional Information

**How to cite this article:** Joseph, N. *et al*. A conformational change within the WAVE2 complex regulates its degradation following cellular activation. *Sci. Rep.* **7**, 44863; doi: 10.1038/srep44863 (2017).

**Publisher's note:** Springer Nature remains neutral with regard to jurisdictional claims in published maps and institutional affiliations.

## Supplementary Material

Supplementary Information
