# Supplementary material for: A conformational change within the WAVE2 complex regulates its degradation following cellular activation
Source: Sci Rep. 2017 Mar 23;7:44863. doi: 10.1038/srep44863 (PMC5362955; doi:10.1038/srep44863)
Supplement: Supplementary Information [file srep44863-s1.pdf]

A conformational change within the WAVE2 complex regulates its degradation following cellular activation

Noah Joseph, Guy Biber, Sophia Fried, Barak Reicher, Omer Levy, Batel Sabag, Elad Noy and Mira Barda-Saad*

The Mina and Everard Goodman Faculty of Life Sciences, Bar-Ilan University, Ramat-Gan 5290002, Israel

Supplementary Information

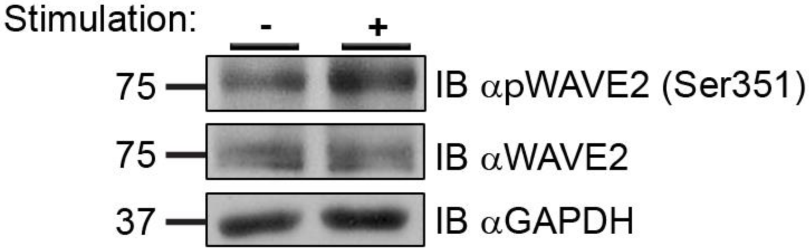

Supplementary Figure S1. Analysis of WAVE2 phosphorylated forms. JVAV cells reconstituted with VAV-1 WT (JVAV/VAV wt) were left unstimulated (-), or were stimulated (+) for 2 min, lysed, and blotted for pWAVE2 (Ser351), WAVE2, and GAPDH (as a loading control).

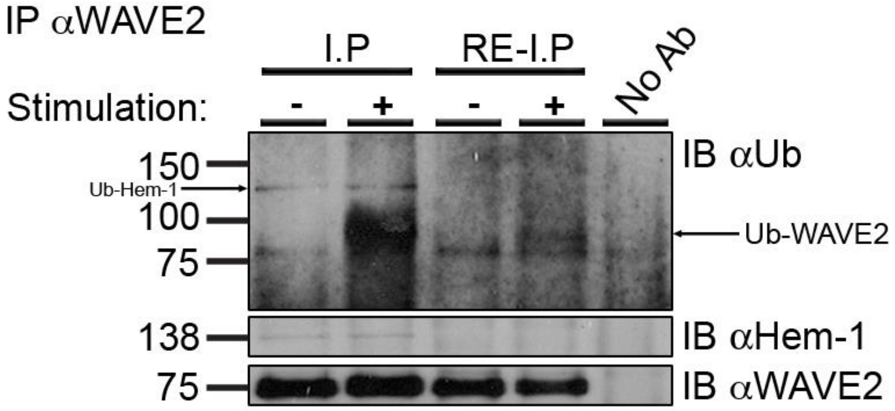

Supplementary Figure S2. WAVE2 is directly ubiquitylated in primary lymphocytes. Human primary peripheral-blood lymphocytes (PBLs) were left unstimulated (-), or were co-stimulated with anti-CD3 and anti-CD28 antibodies (+). Cell lysates were immunoprecipitated using anti-WAVE2 antibody, followed by

boiling in a denaturing buffer to separate WAVE2 associated proteins. After dilution in cell lysis buffer, WAVE2 was re-immunoprecipitated (RE-IP: αWAVE2). The precipitates from the denatured/renatured samples were immunoblotted for ubiquitin, Hem-1 and WAVE2 (IB: αUb, IB: αHem-1 and IB: αWAVE2, respectively). Ubiquitylated endogenous WAVE2 appears as a smear of bands above the molecular weight (MW) of 75 kDa with a prominent band at ~83 kDa. Data shown are representative of two independent experiments.

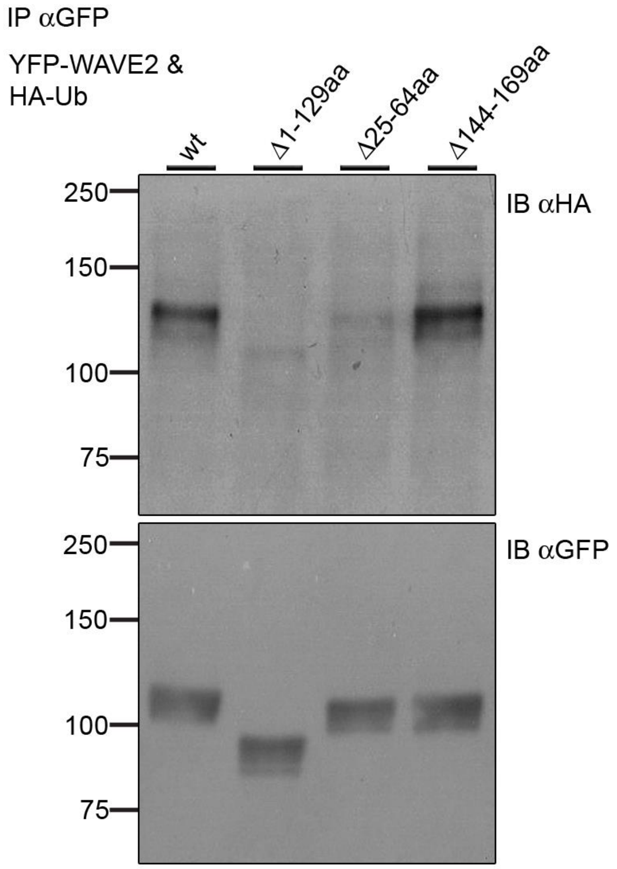

Supplementary Figure S3. Deletion of the first 129aa of WAVE2 leads to a dramatic decrease in its ubiquitylation. 293T cells were co-transfected with constructs encoding HA-tagged ubiquitin together with YFP-WAVE2 wt or with YFP-WAVE2 mutant forms as indicated. YFP-WAVE2 was immunoprecipitated using anti-GFP antibody; complexes were resolved by SDS-PAGE and immunoblotted for ubiquitin using anti-HA antibody, and for YFP-WAVE2 using

anti-GFP antibody. Ubiquitylated YFP-WAVE2 wt appears at a MW of ~110 kDa, which is 8 kDa above the MW of YFP-WAVE2 (~102 kDa).

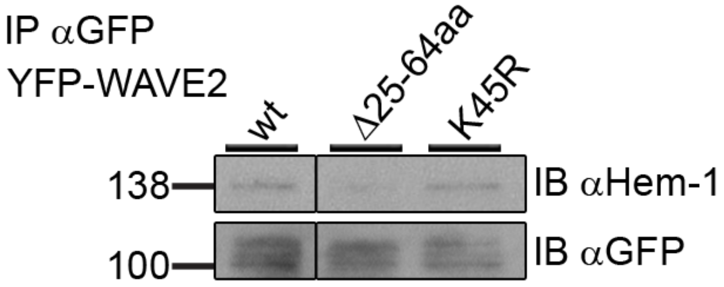

Supplementary Figure S4. The K45R mutation does not impair the WAVE2–Hem-1 interaction. Jurkat T cells expressing YFP-WAVE2 wt or the indicated mutant forms were lysed and subjected to immunoprecipitation with anti-GFP antibody. Samples were analyzed by western blotting with Hem-1 antibody. Vertical lines indicate noncontiguous blots. All lanes are from the same film and exposure time.

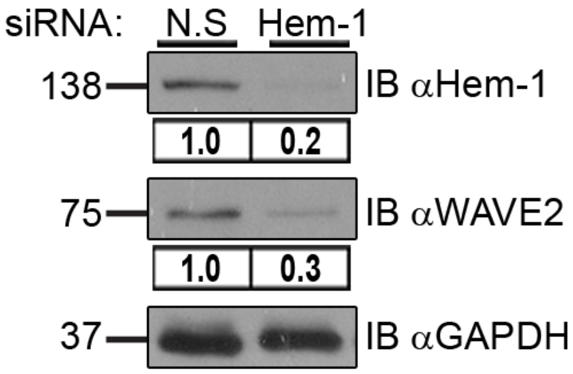

Supplementary Figure S5. Reduction of WAVE2 expression by Hem-1 gene silencing. Jurkat T cells were transfected with siRNA specific to human Hem-1, as well as with a control, non-specific (N.S) scrambled siRNA. After 24 h, cells were co-stimulated with anti-CD3 and anti-CD28 antibodies. Cell lysates were prepared and analyzed for Hem-1 and WAVE2 protein levels. The gene-silencing efficiencies of Hem-1, as well as WAVE2 expression levels, were measured by ImageJ.

Densitometry results for Hem-1 or WAVE2 after normalization relative to the GAPDH values are presented as numbers under each band.

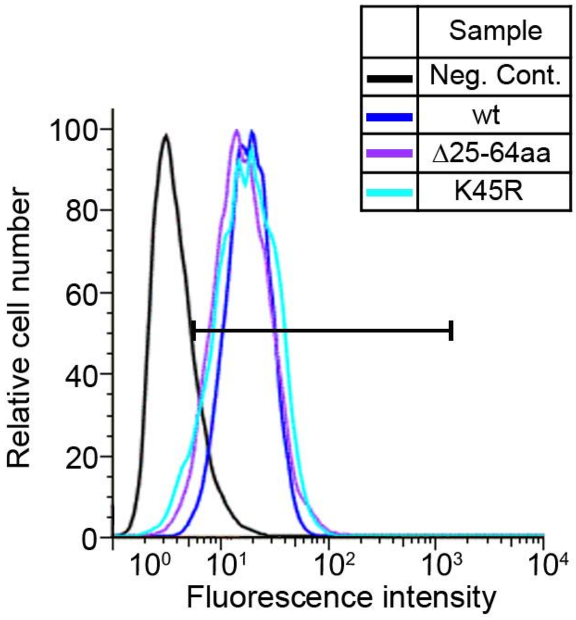

Supplementary Figure S6. Analysis of YFP-WAVE2 expression in sorted cells expressing wt or mutant forms of WAVE2. Jurkat T cells stably expressing YFP-WAVE2 wt, YFP-WAVE2 Δ25-64aa, or YFP-WAVE2 K45R were sorted to obtain cell populations expressing similar protein levels of YFP-WAVE2. The expression levels of YFP-WAVE2 wt and YFP-WAVE2 mutant forms, stably expressed in unstimulated T cells, were confirmed by FACS analysis.

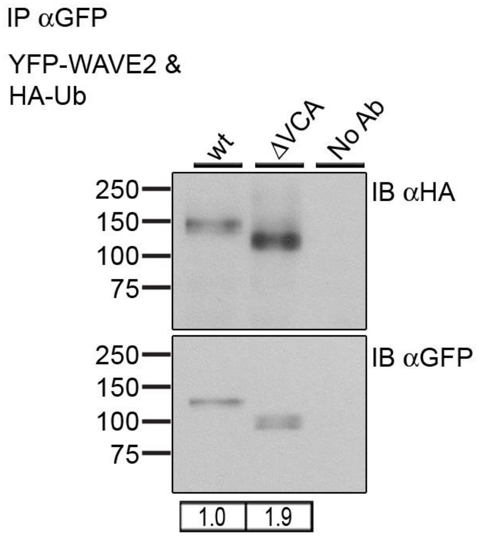

Supplementary Figure S7. Deletion of WAVE2 VCA domain leads to an increase in its ubiquitylation. 293T cells were co-transfected with constructs encoding HA-tagged ubiquitin together with YFP-WAVE2 wt or YFP-WAVE2 ΔVCA. YFP-WAVE2 was immunoprecipitated using anti-GFP antibody. Complexes were resolved by SDS-PAGE and immunoblotted for ubiquitin using anti-HA antibody, and for YFP-WAVE2 using anti-GFP antibody. Ubiquitylated YFP-WAVE2 wt appears at a MW of ~110 kDa, which is 8 kDa above the MW of YFP-WAVE2 (~102 kDa). Densitometric analysis of the bands presented was performed using ImageJ, and normalized relative to the GFP densitometry values.

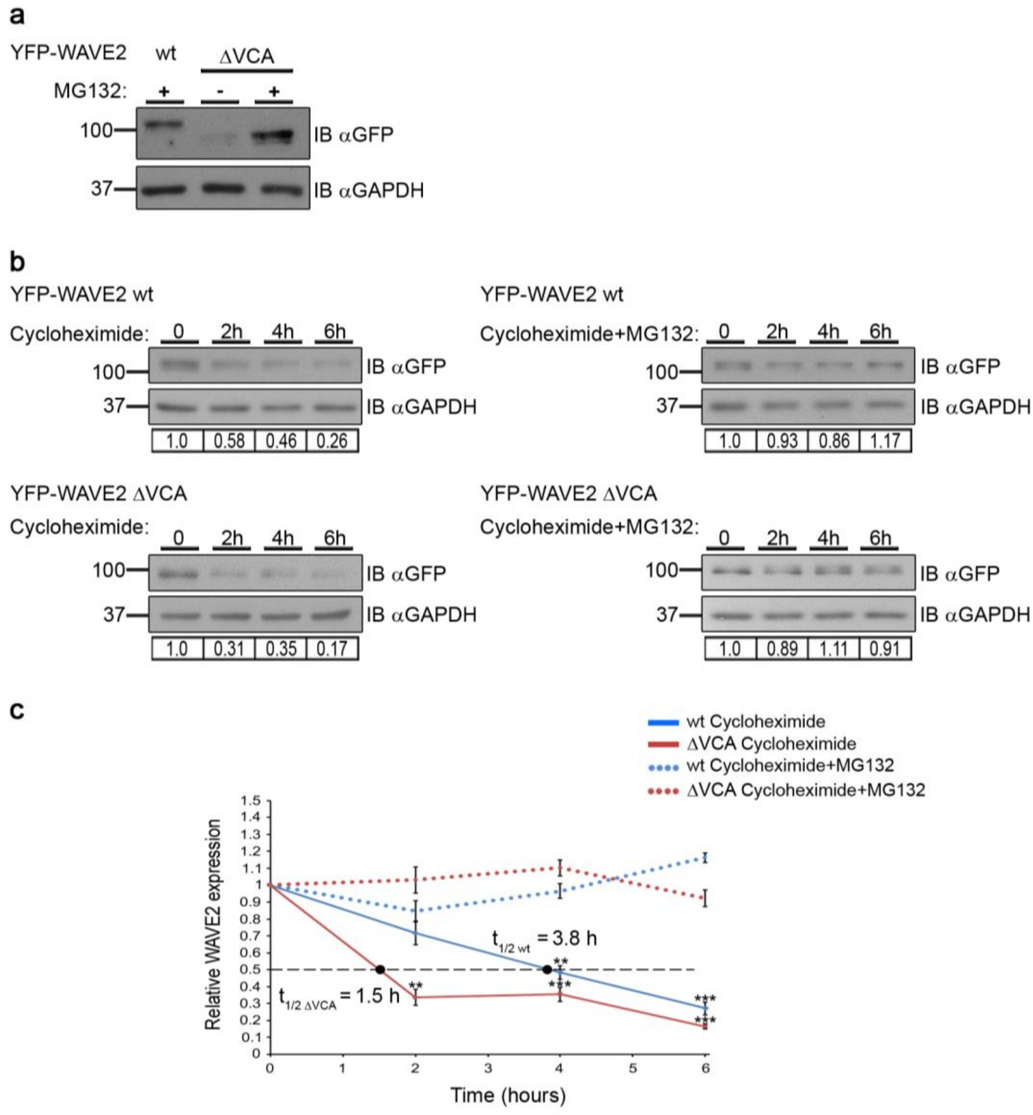

Supplementary Figure S8. Effect of the proteasomal inhibitor, MG132, on the stability of WAVE2 wt and ΔVCA. (**a**) 293T cells were transfected with constructs encoding YFP-WAVE2 wt or YFP-WAVE2 ΔVCA. Cells were left either untreated or pretreated with 50 μM MG132 for 3 h, lysed, and analyzed by immunoblotting. (**b**) 293T cells expressing YFP-WAVE2 wt (upper panels) or YFP-WAVE2 ΔVCA (lower panels) were treated with cycloheximide (0.1 mg/ml) alone to block the protein synthesis (left panels) or with MG132 (50 μM) and cycloheximide (0.1 mg/ml) (right panels). At the indicated time intervals, cell samples were collected and lysed. Lysates were immunoblotted with GFP and GAPDH (loading control) antibodies. Densitometric analyses of the bands presented were performed using ImageJ, and normalized relative to the GAPDH densitometry values. (**c**) Graph summarizing relative levels of WAVE2 (wt and ΔVCA) in cells treated with cycloheximide in the presence or absence of MG132. Calculated half-life of WAVE2 is shown. Data shown are averages of at least three independent experiments. Error bars show SEM. Significance between experimental conditions was determined by two-tailed Student's t-test and is indicated by asterisks (***P*<0.005; ****P*<0.0005).

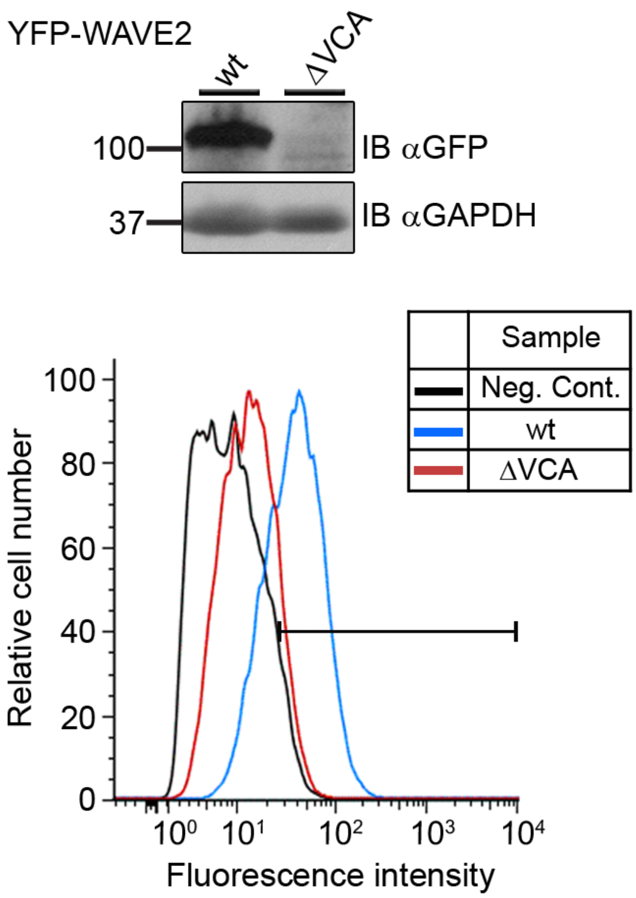

Supplementary Figure S9. The importance of the VCA domain for WAVE2 stability. Jurkat T cells expressing YFP-WAVE2 wt or YFP-WAVE2 ΔVCA were lysed and immunoblotted with anti-GFP (upper panel). The expression levels of YFP-WAVE2 wt and YFP-WAVE2 ΔVCA mutant form were also confirmed by FACS analysis (lower panel). Data shown are representative of at least two independent experiments (upper panel), and five independent experiments (lower panel).

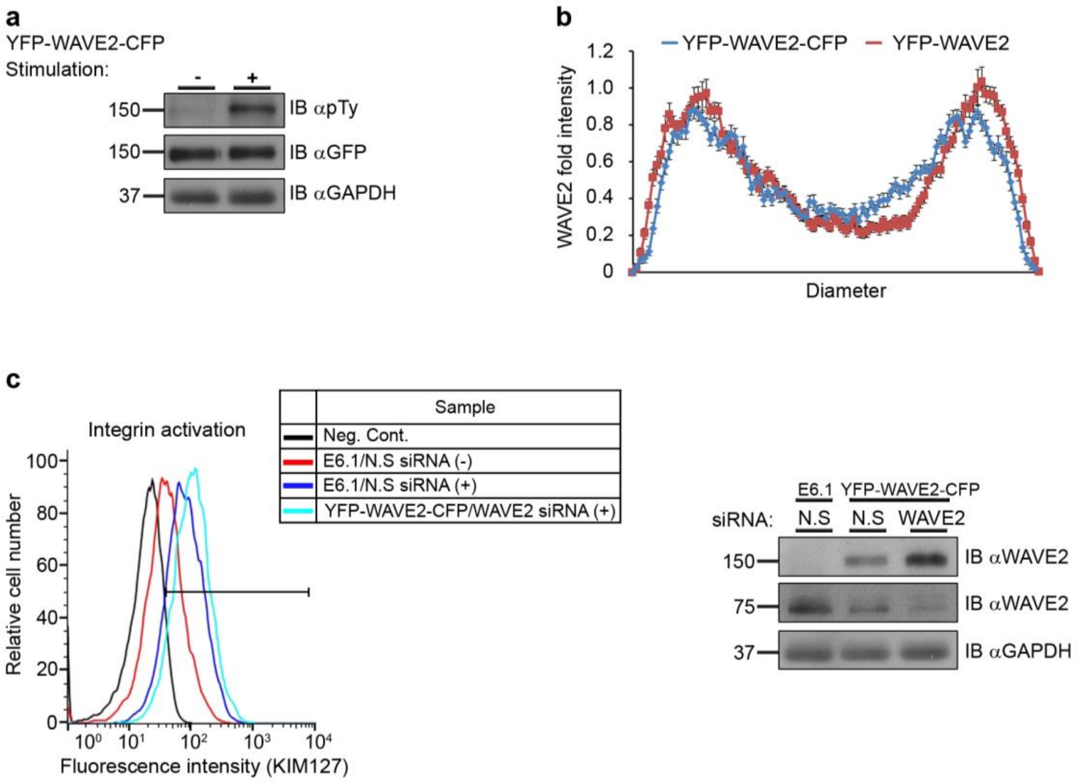

Supplementary Figure S10. Functionality of the YFP-WAVE2-CFP FRET probe. (**a**) Jurkat T cells expressing YFP-WAVE2-CFP were left unstimulated (-), or were co-stimulated with anti-CD3 and anti-CD28 antibodies (+). Cell lysates were immunoblotted for phosphotyrosine (pTy), GFP and GAPDH. (**b**) Cells stably expressing YFP-WAVE2-CFP or YFP-WAVE2 were plated over stimulatory coverslips coated with anti-CD3 antibody. Cells were fixed following 3 min of activation, and imaged by confocal microscopy. YFP-WAVE2-CFP and YFP-WAVE2 distributions in multiple cells were profiled with the use of ImageJ, as described in the Methods section. The plotted average normalized fold intensities of WAVE2 along the diameter of the cells are shown (YFP-WAVE2-CFP, n=40 cells; YFP-WAVE2, n=40 cells). (**c**) Left panel: Flow cytometry analysis of the activated form of LFA-1 expressed in unstimulated (-) or stimulated (+) cells that were transfected with either siRNA specific to endogenous WAVE2 or with N.S siRNA. Flow cytometry plots were gated on total viable cells and, where relevant, on YFP and

CFP fluorescence. Right panel: YFP-WAVE2-CFP expressing T cells transfected with either siRNA specific to endogenous WAVE2 or with N.S siRNA, as well as Jurkat T cells (E6.1) transfected with N.S siRNA, were lysed. The relative abundances of endogenous WAVE2 (75 kD) and exogenous tagged WAVE2 (~150kD) were analyzed by western blotting. In the upper blot, the siRNA-resistant YFP-WAVE2-CFP expression was increased in cells in which endogenous WAVE2 was gene silenced, relative to cells treated with N.S siRNA (lane 3 compared to lane 2, upper blot). This is due to the limited size of the WRC pool, which constitutes the bottleneck limiting the number of WAVE2 proteins. In addition, this results in higher expression of endogenous WAVE2 in Jurkat T cells, relative to that observed in cells expressing YFP-WAVE2-CFP pretreated with N.S siRNA, as the endogenous WAVE2 in Jurkat T cells is free from competition with the transfected form for the WRC proteins.

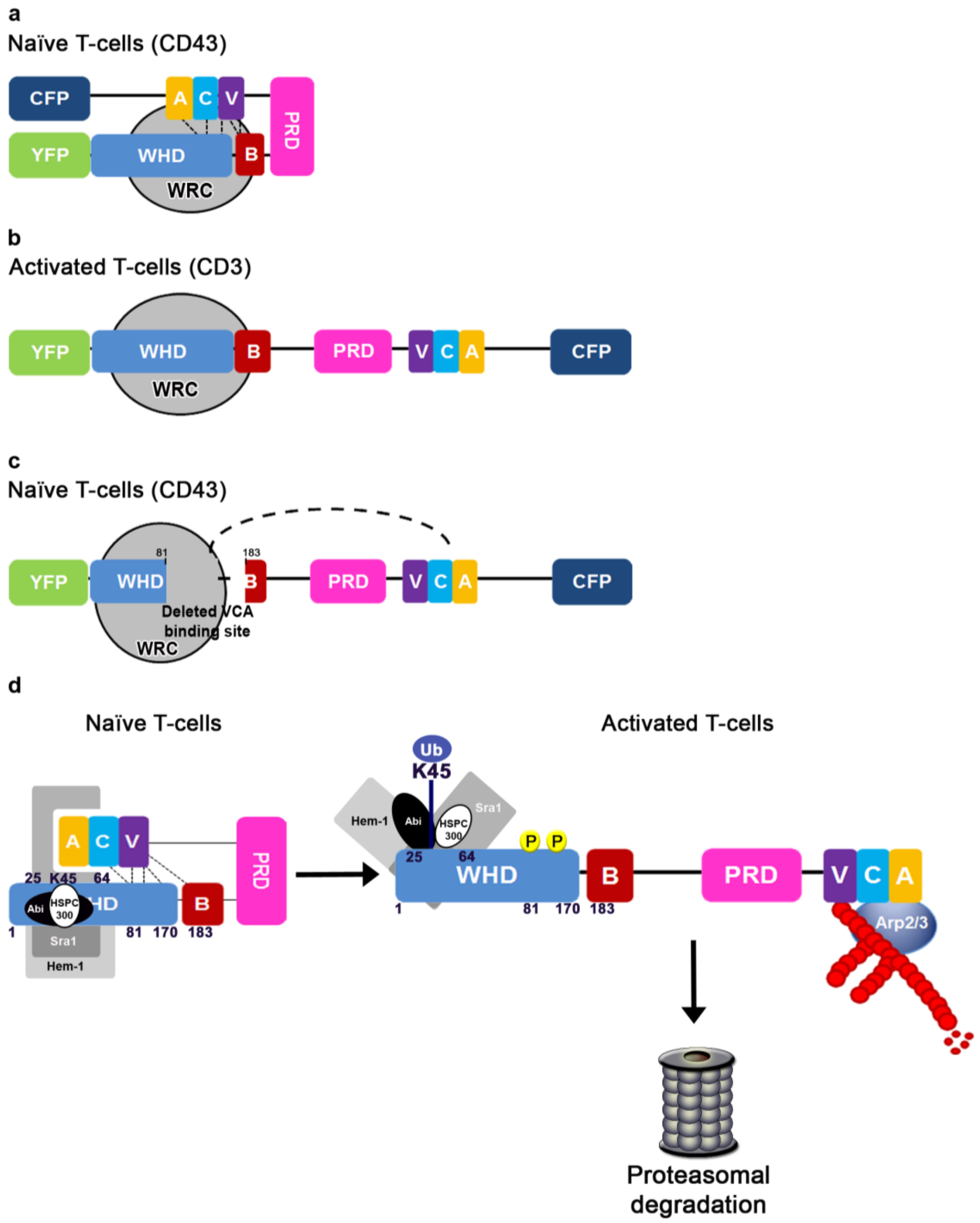

Supplementary Figure S11. Schematic model of WAVE2 autoinhibitiory conformation. (**a**) WAVE2 was tagged with YFP at the N-terminus and with CFP at its C-terminus. Under non-stimulating conditions (anti-CD43), WAVE2 is found in an autoinhibitory conformation facilitated by intra- and inter-molecular interactions between the VCA domain, amino acids 81-183 of WAVE2, and the WAVE regulatory complex (WRC) members. This conformation is indicated by the FRET

measured between CFP and YFP. Disruption of these interactions by either (**b**) TCR activation (anti-CD3), or (**c**) deletion of the VCA binding site within WAVE2 (Δ181-183aa), results in a conformational change to the non-inhibited state, as indicated by the loss of FRET. (**d**) Suggested molecular model of WAVE2 downregulation and the conformational changes that occur within WAVE2 and its regulatory complex following T-cell activation. In naïve T cells, the N terminus of WAVE2 tightly associates with its complex members as well as with the VCA domain through amino acids 25-64, and 81-183, respectively. In addition to its intra-molecular interactions, the VCA domain anchors the WRC to its binding site within WAVE2 (amino acids 25-64). This way, the WRC masks lysine residue 45 of WAVE2, and inhibits its ubiquitylation, enabling WAVE2 accumulation within the T cell. Upon cellular activation, the Rho family GEF, VAV-1, activates Rac1, which binds to Sra1, thereby releasing WAVE2 from its autoinhibitory conformation, and exposing the VCA domain, which can then bind to the Arp2/3 complex, promoting local actin polymerization. In parallel, phosphorylation of WAVE2 occurs, leading to the release of the VCA domain. The interactions between WAVE2 and its complex members are altered following activation, resulting in the exposure of WAVE2 lysine residue 45 to ubiquitylation and to subsequent WAVE2 degradation.
